# The S1 subunits of SARS-CoV-2 variants differentially trigger the IL-6 signaling pathway in human brain endothelial cells and downstream impact on microglia activation

**DOI:** 10.1515/nipt-2023-0024

**Published:** 2024-01-09

**Authors:** Michael Stangis, Daniel Adesse, Bhavya Sharma, Eduardo Castro, Kush Kumar, Neil Kumar, Masha Minevich, Michal Toborek

**Affiliations:** Department of Biochemistry and Molecular Biology, University of Miami Miller School of Medicine, Miami, FL 33136, USA; Laboratory of Structural Biology, Instituto Oswaldo Cruz, Fiocruz, Rio de Janeiro, RJ 21040-360, Brazil

**Keywords:** COVID-19, SARS-CoV-2, neuroinflammation, blood–brain barrier, microglia

## Abstract

**Objectives:**

Cerebrovascular complications are prevalent in COVID-19 infection and post-COVID conditions; therefore, interactions of SARS-CoV-2 with cerebral microvascular cells became an emerging concern.

**Methods:**

We examined the inflammatory responses of human brain microvascular endothelial cells (HBMEC), the main structural element of the blood–brain barrier (BBB), following exposure to the S1 subunit of the spike protein of different SARS-CoV-2 variants. Specifically, we used the S1 subunit derived from the D614 variant of SARS-CoV-2, which started widely circulating in March of 2020, and from the Delta variant, which started widely circulating in early 2021. We then further examined the impact of the HBMEC secretome, produced in response to the S1 exposure, on microglial proinflammatory responses.

**Results:**

Treatment with S1 derived from the D614 variant and from the Delta variant resulted in differential alterations of the IL-6 signaling pathway. Moreover, the HBMEC secretome obtained after exposure to the S1 subunit of the D614 variant activated STAT3 in microglial cells, indicating that proinflammatory signals from endothelial cells can propagate to other cells of the neurovascular unit. Overall, these results indicate the potential for different SARS-CoV-2 variants to induce unique cellular signatures and warrant individualized treatment strategies. The findings from this study also bring further awareness to proinflammatory responses involving brain microvasculature in COVID-19 and demonstrate how the surrounding microglia react to each unique variant derived response.

## Introduction

Coronavirus disease-19 (COVID-19), caused by severe acute respiratory syndrome coronavirus 2 (SARS-CoV-2) and originally reported in December 2019, continues to persist despite extensive vaccination efforts. COVID-19 can be a devastating disease as it can, in serious cases, lead to organ failure, pneumonia, and death [1]. Moreover, neurological and cerebrovascular complications from the Central Nervous System (CNS), such as stroke and encephalopathy, are prominent in hospitalized cases [2], [3], [4], [5]. Our group has provided evidence that cells of the neurovascular unit (NVU) express a receptor profile that is necessary for SARS-CoV-2 entry into host cells [6]. In addition, we, and others, demonstrated that exposure of primary human brain microvascular endothelial cells (HBMEC) to the S1 subunit of the SARS-CoV-2 spike (S) protein resulted in a decrease in tight junction protein expression [6, 7]. SARS-CoV-2 has since been found to be able to infect cells of the CNS [8, 9], which can lead to neurological complications [10, 11].

COVID-19 has persisted due to the ability of SARS-CoV-2 to mutate into more infectious subvariants. For example, mutations within the S protein of the Delta variant conferred a greater binding affinity to the ACE2 receptor, allowing for a higher rate of infection and viral load compared to earlier SARS-CoV-2 variants [12]. The Delta variant remained the dominant variant until the end of 2021, when the omicron variant spread rapidly. Additional mutations in the S protein of the omicron variant conferred it advantages in infectivity, and with over 50 mutations made it more likely to escape neutralization by mRNA vaccines [13]. Nevertheless, infection with the omicron variant has been shown to produce less severe symptoms when compared to the Delta variant [14, 15].

In the present study, we compared the responses of HBMEC to the S1 subunits of the SARS-CoV-2 S protein of the early circulating D614 variant and the Delta variant. We focused on the interleukin 6 (IL-6) pathway, an inflammatory cytokine that plays a key role in acute phase response and host defense to pathogens [16], [17], [18], and is released as part of SARS-CoV-2-induced inflammatory cytokine storm [19], [20], [21]. Additionally, we evaluated if activation of brain endothelial cells can be propagated to other cell types of neurovascular unit. Specifically, we assessed how microglia respond to the HBMEC secretome following exposure to the S1 subunits of the S protein as a downstream effect, as microglia contribute to BBB dysregulation and local inflammation [22, 23].

Our results indicate that exposure of the S1 subunit of the S protein of the D614 SARS-CoV-2 variant induces a stronger IL-6 response compared to the Delta variant in HBMEC, manifested as an increase in IL-6 mRNA level and IL-6 release, alongside enhanced expression of phosphorylated signal transducer and activator of transcription 3 (STAT3). Exposure of microglia to the conditioned media of HBMEC yielded similar patterns, with the D614 SARS-CoV-2 variant inducing enhanced STAT3 translocation to the nucleus. These results indicate that SARS-CoV-2 variants induce differential responses as determined in part by the neuroinflammatory pathways that S protein triggers in BBB cells. These effects may lead to cerebrovascular dysregulation and comorbidities.

## Materials and methods

### Cell culture, maintenance, and experimental design

Primary human brain microvascular endothelial cells (HBMEC; Cell Systems, #ACBRI 376) were seeded and cultured in a Complete Classic Medium with Serum and CultureBoost™ (Cell Systems, 4Z0-500). Following the first two passages, cultures were kept in a 1:1 mixture of the Complete Classic Medium and Complete Serum-Free Medium Kit with RocketFuel™ (Cell Systems, SF-4Z0-500). Two days after seeding for experiments, the medium was changed, with the new ratio of Classic to Serum-free media being 25–75 %. Two days after, the medium was changed to 100 % Serum-free medium and all experiments were performed in serum-free medium. Cells were exposed to the S1 subunit of the S protein from either the D614 variant (Ray Biotech, 230-01101) or Delta variant (ACROBiosystems, NC1980868) at 15 nM.

To establish the S1 protein level employed in the present study, we performed preliminary experiments on the impact of the S1 protein on tight junction protein expression in HBMEC at concentrations ranging from 11.2 nM up to 100 nM. The S1 protein at 11.2 and 15 nM [6] reduced the expression of occludin at comparable levels of higher concentrations. Similar levels of the S protein from the SARS 2003 epidemic were reported to have proinflammatory effects [24]. Moreover, this dosage was in tune with studies from other groups [6, 25, 26]; however, it was higher that the levels of the S1 protein in plasma samples of COVID-19 patients, which was up to 100 pg/ml [27]. Heat-treated forms of these proteins were generated by heating for 5 min at 95 °C. Lipopolysaccharides (LPS; Millipore Sigma L4391) derived from *E. coli* was administered at 100 ng/mL. Control cells were exposed to vehicle (phosphate buffered saline; PBS). Treatments were reapplied every 24 h. For cultures exposed for 72 h, media was exchanged after 48 h.

HMC3 (immortalized microglia cells; ATCC, CRL-3304™) were cultured in Eagle’s Minimum Essential Medium (EMEM, ATCC 30-2003) supplemented with 10 % Fetal Bovine Serum (FBS, ATCC 30-2020). The medium was changed every two days. For the experiments, HMC3 were seeded into 6-well plates at a density of 300,000 cells per well. Two days after seeding, the medium was replaced with one mL of HBMEC conditioned media for 15 min or 6 h. Additional controls were performed by treating HCM3 with LPS 100 ng/mL or 350 pg/mL of human recombinant IL-6 (ThermoFisher, #PCH0065).

### Enzyme-linked immunosorbent assay (ELISA)

Following treatments, the medium from each well was collected, centrifuged to remove any cell debris, and kept at −20 °C until analysis or future use with HMC3 microglia. The levels of IL-6 were assessed by Human ELISA Kit (ABCAM, ab178013) according to the manufacturer’s instructions.

### RT-qPCR

HBMEC cultures were washed with PBS, RNA was harvested using the RNeasy Mini Kit (Qiagen, 74,104), and quantified with Nanodrop (ThermoFisher). Then, 100 ng of RNA diluted in 1 μL of RT-PCR Grade Water (ThermoFisher Scientific, AM9935) was used for each reaction, with 7 μL of RT-PCR Grade Water, 10 µL qScript™ XLT One-Step RT-qPCR ToughMix^®^ (VWR, 89,236-676) and 1 μL of the corresponding primer set for the following target gene amplification (all from ThermoFisher Scientific): IL-6 (Hs00174131_m1), IL-6R (Hs01075664_m1), glycoprotein130 (Ggp130)/IL-6 signal transducer (IL6ST) (Hs00174360_m1), and STAT3 (Hs00374280_m1). Eukaryotic 18S rRNA Endogenous Control (ThermoFisher Scientific, ref #4319413E) was used as the housekeeping gene. All samples were assayed in technical duplicates and relative expression to untreated controls was determined by the 2^−ΔΔct^.

### Immunoblotting

HBMEC cultures were washed with PBS, and protein was harvested by lysing the cells with Radio Immuno Precipitation Assay (RIPA) buffer containing Complete Mini Protease Inhibitor Cocktail (Roche). HMC3 were washed with PBS, with additional steps performed to isolate the nuclear and cytoplasmic fractions as described earlier [28].

Protein concentrations were quantified using a BCA Protein Assay Kit (ThermoFisher Scientific, ref #23223) and immunoblotting was performed as described previously [6]. The following primary antibodies were used: anti-gp130 (IL6ST; Abcam, ab283685), anti-IL-6R (Abcam, ab128008), anti-phosphorylated STAT3 (Tyr705, Cell Signaling, 9145T), and anti-STAT3 (Cell Signaling, 124H6). In addition, the following secondary antibodies (all from Licor) were applied: IRDYE^®^ 680RD (926-68073 + 926-68072) and IRDYE^®^ 800CW (926-32212 + 926-32211).

Protein levels were normalized to anti-glyceraldehyde 3-phosphate dehydrogenase (GAPDH, Novus, NB3000-221R) for total and cytoplasmic fractions, and to anti-Lamin A/C (Cell Signaling, #4777S) for nuclear fractions. Membranes that were used to read multiple proteins were stripped using NewBlot Nitro Stripping Buffer (Licor, ref #928-40030) for 10 min, then the steps were repeated starting with the blocking of the membrane.

### Statistical analysis

Western blotting and RT-qPCR data were analyzed from a minimum of three independent experiments. Normalized values for all samples were compared to their respective controls. Statistical analyses were performed on GraphPad Prism, for each time point we performed Two-Way ANOVA with Bonferroni post-tests. Values of p<0.05 were considered statistically significant. All data are expressed as mean values ± standard errors of mean (SEM).

## Results

### Spike S1 protein from different SARS-CoV-2 variants induce distinct IL-6 pathway activation levels in HBMEC

**Figure 1:**
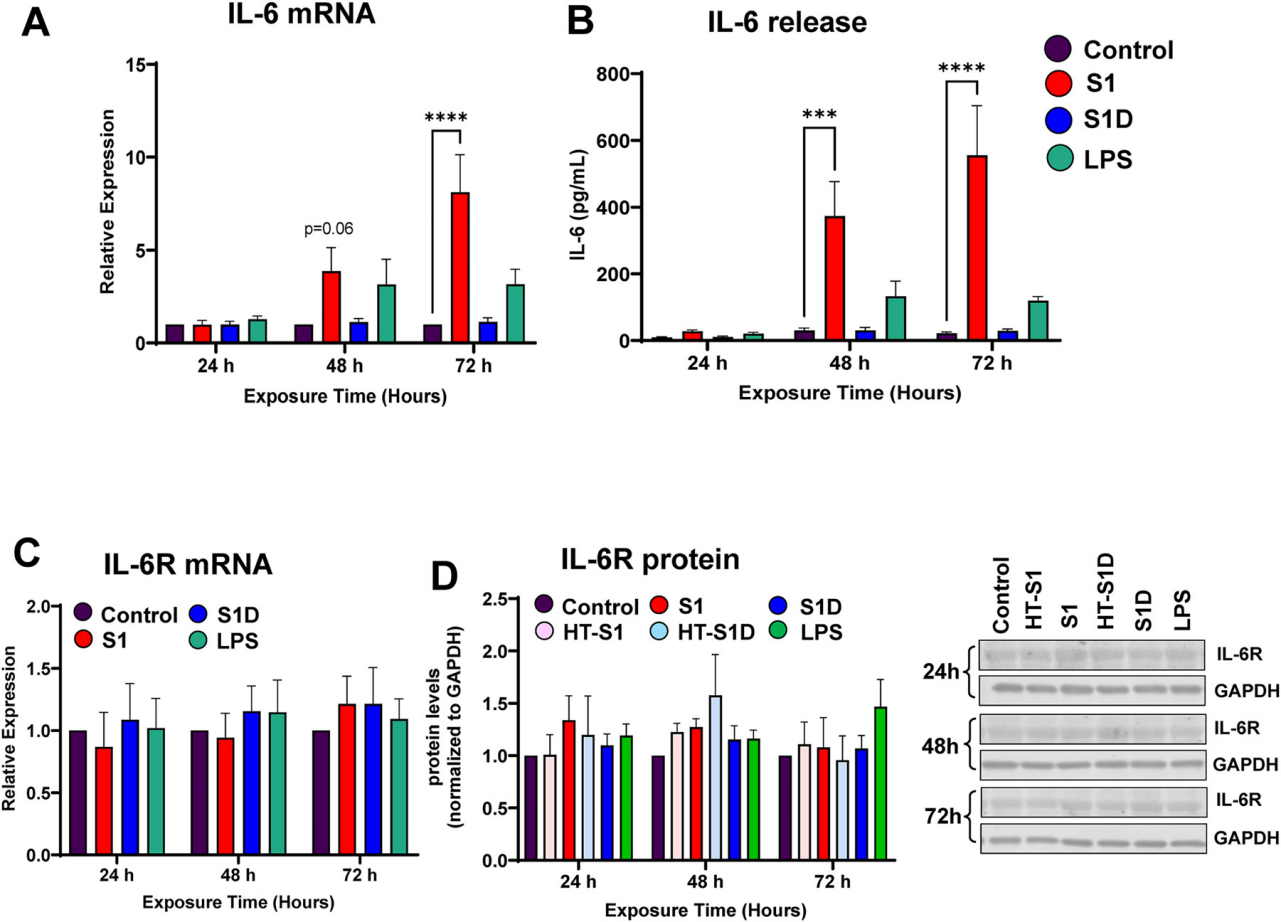
The S1 subunit of the spike protein differentially induced IL-6 secretion by brain endothelial cells. Exposure to the S1 subunit of the S protein of the D614 variant resulted in an upregulation of IL-6 mRNA expression (A) and IL-6 release into cell culture media (B). No significant changes were observed in IL-6 receptor (IL-6R) mRNA (C) or protein (D) expression. (D) left panel, quantitative data; right panel representative blots for IL-6R. ^*^p<0.05; ^**^p<0.01; ^***^p<0.001; ^****^p<0.0001. Two-way ANOVA with Bonferroni post-test. Data represents means ± SEM of five independent experiments.

Expression and release of IL-6, a pro-inflammatory cytokine commonly found in the cytokine storm associated with acute COVID-19 [18], [19], [20], were analyzed in HBMEC upon exposure to the S1 subunit of the S protein from the early D614 variant (S1) and from the Delta variant (S1D). Treatment with LPS served as the positive control in these experiments. Exposure to S1 for 72 h significantly increased IL-6 mRNA levels as compared to vehicle controls. While the exposure for 48 h induced a strong tendency to elevate IL-6 mRNA levels, these changes did not reach statistical significance. Interestingly, a shorter exposure to S1 and treatment with S1D did not affect IL-6 mRNA levels, indicating specificity of responses (Figure 1A). Consistent with the gene expression results, exposure to S1 for 48 and 72 h resulted in a remarkable increase in IL-6 release into cell culture media. Conversely, exposure to S1D did not affect IL-6 protein levels (Figure 1B).

After observing that S1 subunits induced differential IL-6 production, we then evaluated whether the downstream IL-6 signaling pathway is affected in S1-treated HBMEC cultures. IL-6 binds to IL-6 receptor (IL-6R) and co-receptor gp130, which leads to the downstream activation of the Janus kinase (JAK)-STAT3 signaling pathway [17], followed by transcription of cytokines and growth factors involved in inflammatory and cell cycle processes [18]. No significant changes were observed in IL-6R mRNA (Figure 1C) or protein (Figure 1D) levels across all the treatments at the studied timepoints. In Figure 1D, the left panel reflects quantitative results of IL-6R protein, while the right panel illustrates representative blots. We also observed no impact of employed treatments on the levels of soluble IL-6R (data not shown), also an inducer of trans-activation of JAK-STAT signaling pathway [18].

### D614 spike S1, but not Delta S1, induces STAT3 pathway activation in HBMEC

**Figure 2:**
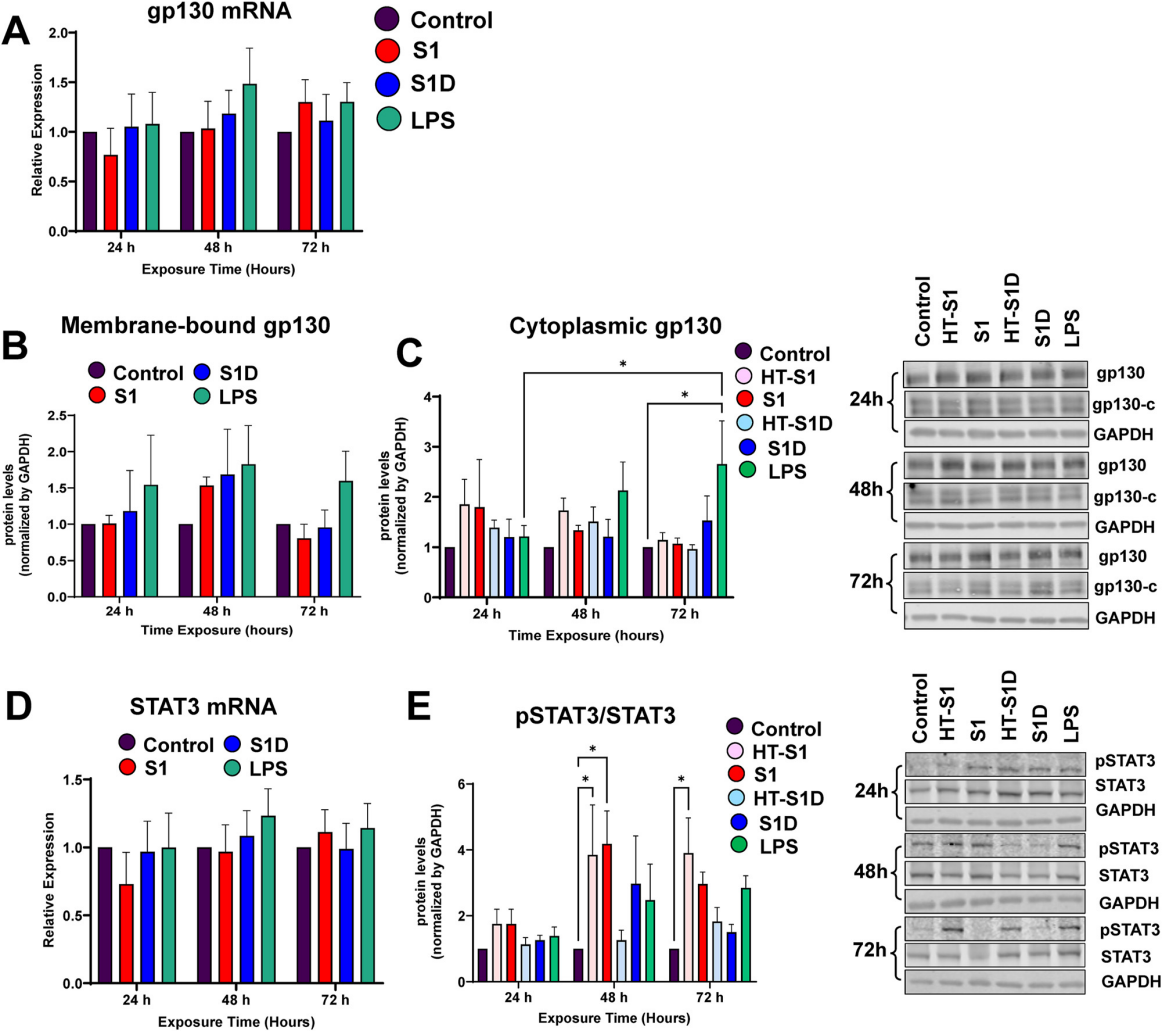
The S1 subunit of the spike protein selectively induced STAT3 activation in brain endothelial cells. gp130 mRNA expression revealed a trend towards an increase in the S1-treated HBMEC at 72 h (A). A similar increasing trend in the membrane-bound (B) or cytoplasmic (C) gp130 occurred across all treatment groups. The impact of LPS (positive control) on the cytoplasmic fraction was significant. (D) STAT3 mRNA expression was not changed in all employed experimental groups. (E) Treatment with S1 for 48 h and with heat-inactivated (HT)-S1 for 48 and 72 h significantly increased the ratio of phosphorylated STAT3 (pSTAT3) to total STAT3. (C and E), left panels, quantitative immunoblotting data, right panels, representative blots. ^*^p<0.05; ^**^p<0.01; ^***^p<0.001; ^****^p<0.0001, two-way ANOVA with Bonferroni post-test. Data represents means ± SEM of five independent experiments.

We next evaluated the impact of S1 and S1D treatment on the expression profile of gp130, a transmembrane adaptor protein, which is involved in the signaling of other members of the IL-6 cytokine family, such as cardiotrophin, IL-11 and IL-27 [29]. mRNA expression of gp130 remained unaltered in HBMEC treated with S1 or S1D, despite a tendency to be increased at 72 h upon exposure to S1 (Figure 2A).

We then assessed the expression of the membrane-bound and cytoplasmic fractions of gp130. The rationale of these experiments was related to the fact that gp130 is ubiquitously endocytosed and relates to downstream IL-6 signal transduction [17]. While there were trends towards an increase in the levels of the membrane-bound gp130 in all treatment groups at 48 h, these changes did not reach statistical significance (Figure 2B). Similarly, treatment for 24 h or 72 h also did not induce the expression of membrane-bound gp130 levels (Figure 2B).

The cytoplasmic fraction of gp130, which is phosphorylated by JAK following dimerization with the IL-6/IL-6R complex to allow binding of STAT3 and activation of the JAK-STAT3 pathway [17, 27], was also quantified by immunoblotting. The results indicated strong trends towards upregulation of cytoplasmic gp130 after 48 h of exposure to S1, heat-inactivated S1 (HT-S1), or S1D (Figure 2C). Exposure for 24 h to S1 and HT-S1 also resulted in a strong trend toward an increase in the expression of cytoplasmic gp130. In Figure 2C, the left panel reflects quantitative results on IL-6R protein, and the right panel illustrates representative blots.

Activation of the JAK-STAT3 pathway is a downstream effect of IL-6 signaling and leads to a variety of cellular responses [16, 17]. This pathway is executed by the initial phosphorylation of STAT3 (pSTAT3) by gp130, following the phosphorylation of JAK [17, 18, 29]. pSTAT3 then translocates to the nucleus, where it binds to DNA and regulates gene transcription. Therefore, we evaluated STAT3 mRNA and STAT3 phosphorylation level. While STAT3 mRNA levels did not alter upon the employed treatment (Figure 2D), there were significant differences in the levels of pSTAT3 between the treatment groups (Figure 2E). Exposure to S1 and HT-S1 for 48 h markedly upregulated the ratio of pSTAT3/STAT3 as compared to control, indicating activation of the STAT3 pathway. In addition, treatment for 24 h or 72 h with S1 showed a strong trend towards an increase of pSTA3/STAT3. There was also a trend toward an increase in STAT3 phosphorylation in S1D-treated cells at 48 h. In Figure 2E, left panel reflects quantitative results on IL-6R protein, and right panel illustrates representative blots.

### Impact of HBMEC conditioned media on HMC3 microglial cells

**Figure 3:**
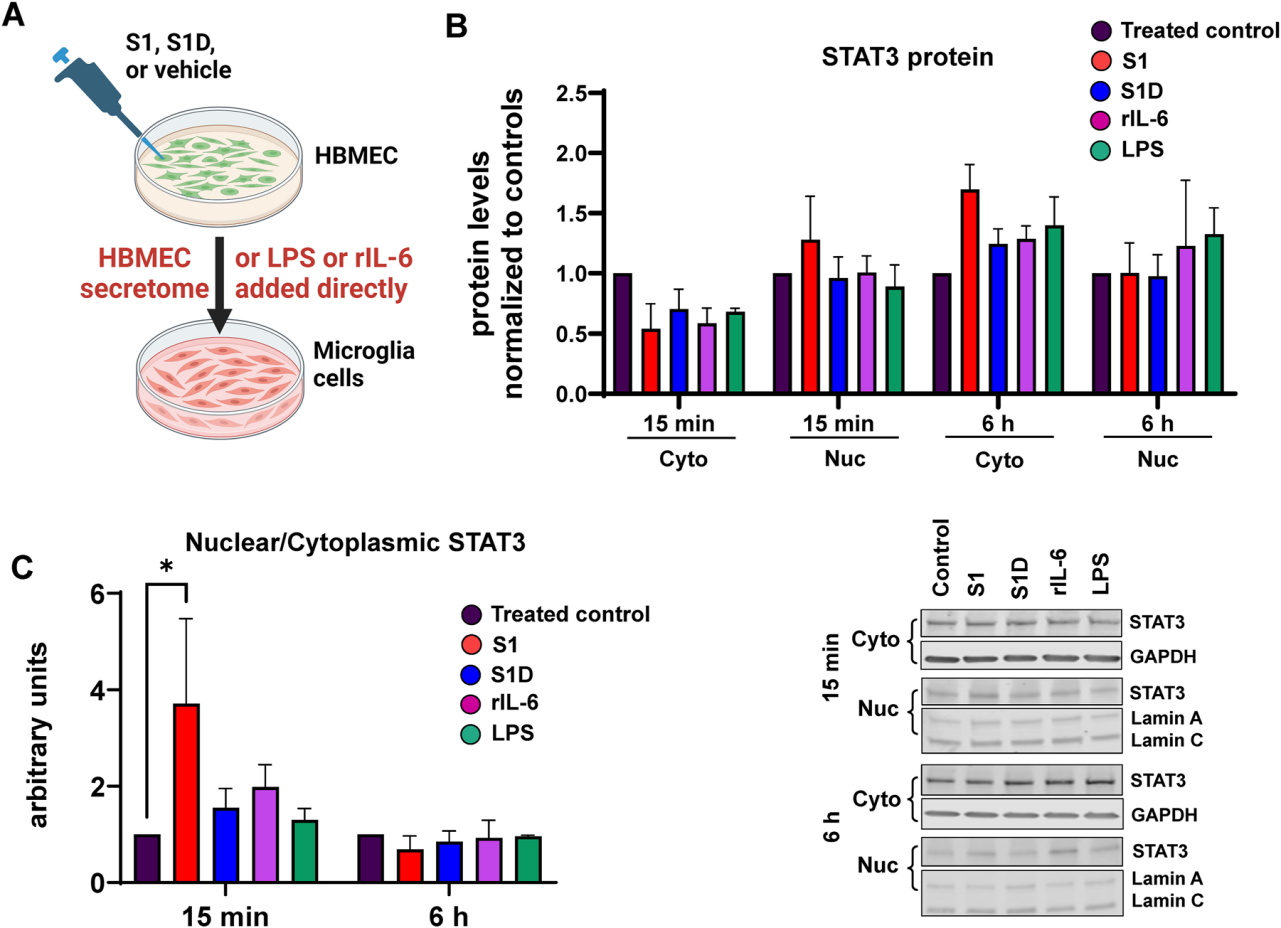
Secretome from the S1 subunit-treated brain endothelial cells induce STAT3 nuclear translocation in human microglial cells. (A) Experimental approaches: HBMEC were treated with S1 or S1D. Then, HMC-3 microglial cells were exposed for 15 min or 6 h to conditioned media obtained from HBMEC or to directly recombinant human IL-6 (rIL-6) or LPS. (B) No significant changes in STAT3 expression were observed in either nuclear (Nuc) or cytoplasmic (Cyto) fractions across the timepoints and the treatment in HMC-3 microglial cells. Left panel, quantitative results, right panel, representative immunoblots. GAPDH was used as loading control for cytoplasmic fractions and Lamin A/C for nuclear fractions. (C) Exposure to conditioned media from S1-treated HBMEC resulted in a significant increase in nuclear/cytoplasm STAT3 ratio in HMC-3 cells after 15 min of treatment, as compared to controls. ^*^p<0.05, two-way ANOVA with Bonferroni post-test. Data represents means ± SEM of four independent experiments.

Based on the observation that HBMEC selectively release IL-6 in response to treatment with D614 S1 protein, followed by STAT3 phosphorylation, we next evaluated whether this event could trigger a secondary response in microglia. Microglia are CNS-resident immune cells and are capable of producing inflammatory mediators, including those found in COVID-19 induced cytokine storm [18], [19], [20]. We exposed HMC3 microglia to conditioned media obtained from 48 h-treated HBMEC. In addition, treatment with LPS or recombinant human IL-6 (350 pg/mL) for 15 min or 6 h were used as positive controls (Figure 3A). The experiments focused on STAT3 translocation into the nucleus as an indicator if S1 protein-exposed endothelial cells can propagate a proinflammatory signal. STAT3 is a downstream target of the IL-6 cytokine family and acts as a transcription factor, leading to the activation of inflammatory gene expression [30].

When measuring STAT3, there were trends toward an increase in expression in the cytoplasmic fraction after 6 h of exposure to the conditioned media in all groups (Figure 3B). The most pronounced trends in elevated STAT3 expression were in the nuclear fraction 15 min and the cytoplasmic fraction 6 h after exposure to the S1 HBMEC secretome. We then compared the ratio of STAT3 localization in the nuclear versus cytoplasmic fractions of HMC3 as recruiting STAT3 to the nucleus is required for its transcriptional activity. At 15 min all treatment groups showed an increased proportion of STAT3 in the nucleus than the control group, with the S1 HBMEC secretome having the most pronounced and statistically significant impact (Figure 3C). In Figure 3C, the left panel reflects quantitative results, and the right panel illustrates representative blots. The results were transient and little differences were seen across all the treatment groups at 6 h.

Interestingly, the impact of the S1 HBMEC secretome was more pronounced that treatment with recombinant IL-6 alone, suggesting that IL-6 present in conditioned media is not solely responsible for changes in STAT3 localization. Thus, additional factors induced by the exposure of the S1 subunits to HBMEC likely contribute to activation of HMC3 microglia.

## Discussion

Cerebrovascular comorbidities have been identified in ∼10 % of COVID-19 survivors within 6 months post infection, indicating their importance for the development of post-COVID conditions [31]. Moreover, disruption of the BBB as determined by elevated albumin levels in the cerebral spinal fluid [32] and increased fibrinogen deposition in the brains, appears to be common in COVID-19 patients [33]. To support these findings, elevated levels biomarkers of vascular injury, such as soluble intercellular adhesion molecule 1 (sICAM-1) or vascular cell adhesion molecule 1 (sVCAM-1), and markers of BBB damage, such as S100β, have been described in the plasma of COVID-19 patients [34]. Moreover, inflammatory changes involving the brain endothelium have been described in autopsies of human samples with COVID-19 [33]. Further dysregulation of the BBB in COVID-19 patients [35, 36] suggests that SARS-CoV-2 can cross the BBB. While this has yet to be demonstrated in patients, it has been modeled in mice [37].

The finding of the involvement of cerebrovascular system in COVID-19 physiopathology raised a possibility that SARS-CoV-2 can directly infect brain endothelial cells and possibly other cells of the NVU, which form the BBB. We have previously shown that cells of the NVU express the known receptors required for SARS-CoV-2 to enter host cells, including ACE2 and TMPRSS2 [6]. However, several laboratories, including our group, failed to demonstrate any productive SARS-CoV-2 replication in brain endothelial cells [38], [39], [40]. While a productive infection has recently been reported in human induced pluripotent stem cell-derived brain capillary endothelial-like cells, this was achieved only after employing a high multiplicity of infection (MOI) 10, which questions the pathological relevance of these findings [41]. The current consensus is that brain endothelial cells can be activated, without the virus entering the cells, which provides a rationale for the current study design in which the S1 subunit of the S protein has been employed. As the virus continues to mutate and adapt, it is increasingly important to understand the effects that distinct SARS-CoV-2 variants may have on the brain. Moreover, COVID-19 vaccines use the S protein as an immunogen [42] and the protein is capable of crossing the BBB [43], which further justifies the relevance and importance of studying how viral proteins interact with human cells.

Our experiments focused on the impact of the S1 protein on IL-6 levels and IL-6 signaling. IL-6 is one the major inflammatory molecules present in the cytokine storm response in COVID-19 patients, and we have demonstrated that HBMEC exposed to SARS-CoV-2 also display increased IL-6 expression. IL-6 is a key cytokine in BBB integrity and is a hallmark of neuroinflammatory processes [44]. Our group published recently that immortalized HBMEC exposed to SARS-CoV-2 have increased IL-6 gene expression [38]. The results of the current study indicate that treatment of HBMEC with the S1 subunit markedly upregulated the release of IL-6. These results are consistent with observations that exposure to this protein can induce dysregulation of tight junction proteins and endothelial activation [6, 7, 45]. They also reinforce the notion that viral proteins are capable of stimulating an innate inflammatory response in BBB cells, without a productive infection. Interestingly, there were also substantial differences in IL-6 upregulation between variants of the S1 subunit, with the D614 variant inducing more pronounced effect as compared to the Delta variant. These effects translated to a stronger downstream response as indicated by an increase in phosphorylation of STAT3 in cells exposed to the S1 subunit from the D614 variant. These findings align with clinical data that the Delta variant induces overall more severe symptoms and outcomes; however, the D614 variant may more strongly impact the brain [46]. Indeed, the wild type virus and the Omicron variant may have a higher potential for neurological damage due to their ability to induce CNS cell stress, affect extracellular glutamate concentration, and damage BBB cellular components. In contrast, the Eta, Delta, Beta, and Alpha variants seem to have comparatively lower impact on neurological health, as they either do not cause direct cellular death, do not affect glutamate concentration significantly, or do not induce BBB breakdown [46].

Taking into consideration the important role of the BBB as the major barrier protecting the brain against blood-borne pathogens, we evaluated whether activation of brain endothelial cells by the S1 subunit can trigger inflammatory events in other cells of the NVU. Indeed, HMC3 microglia cells exposed to the secretome of HBMEC induced by the S1 subunit from the D614 variant resulted in enhanced rate of STAT3 translocation to the nucleus, which is indicative of STAT3 activation. These findings imply that direct passage of SARS-CoV-2 into the brain parenchyma is not necessary to illicit an inflammatory response in the NVU. This provides another possible avenue for the neurological effects that have been reported in COVID-19 patients outside of the virus passing through the BBB to interact directly with the cells of the CNS [47], [48], [49].

In conclusion, the S1 subunit of the S protein of the D614 variant of SARS-CoV-2 and the Delta variant differentially induce IL-6 release and phosphorylation of STAT3 in HBMEC. The secretome of HBMEC following exposure to the S1 subunit of the D614 variant can increase STAT3 signaling in microglia. These responses suggest that SARS-CoV-2 variants induce unique cellular signatures and warrant individualized treatment strategies. Additionally, an active infection of the NVU and/or direct exposure to SARS-CoV-2 via passing through the BBB do not appear to be necessary for the induction of inflammatory responses. These findings bring additional awareness to proinflammatory responses involving brain microvasculature in COVID-19 and its downstream effects on cells of the NVU.
